# How Does Rice Defend Against Excess Iron?: Physiological and Molecular Mechanisms

**DOI:** 10.3389/fpls.2020.01102

**Published:** 2020-08-07

**Authors:** May Sann Aung, Hiroshi Masuda

**Affiliations:** Department of Biological Production, Faculty of Bioresource Sciences, Akita Prefectural University, Akita, Japan

**Keywords:** iron excess, rice, OsNAS3, HRZ, OsVIT2, ROS, iron homeostasis, tolerant mechanism

## Abstract

Iron (Fe) is an essential nutrient for all living organisms but can lead to cytotoxicity when present in excess. Fe toxicity often occurs in rice grown in submerged paddy fields with low pH, leading dramatical increases in ferrous ion concentration, disrupting cell homeostasis and impairing growth and yield. However, the underlying molecular mechanisms of Fe toxicity response and tolerance in plants are not well characterized yet. Microarray and genome-wide association analyses have shown that rice employs four defense systems to regulate Fe homeostasis under Fe excess. In defense 1, Fe excess tolerance is implemented by Fe exclusion as a result of suppression of genes involved in Fe uptake and translocation such as *OsIRT1*, *OsYSL2*, *OsTOM1*, *OsYSL15*, *OsNRAMP1*, *OsNAS1*, *OsNAS2*, *OsNAAT1*, *OsDMAS1*, and *OsIRO2*. The Fe-binding ubiquitin ligase, HRZ, is a key regulator that represses Fe uptake genes in response to Fe excess in rice. In defense 2, rice retains Fe in the root system rather than transporting it to shoots. In defense 3, rice compartmentalizes Fe in the shoot. In defense 2 and 3, the vacuolar Fe transporter *OsVIT2*, Fe storage protein ferritin, and the nicotinamine synthase *OsNAS3* mediate the isolation or detoxification of excess Fe. In defense 4, rice detoxifies the ROS produced within the plant body in response to excess Fe. Some *OsWRKY* transcription factors, *S-nitrosoglutathione-reductase* variants, p450-family proteins, and *OsNAC4*, *5*, and *6* are implicated in defense 4. These knowledge will facilitate the breeding of tolerant crops with increased productivity in low-pH, Fe-excess soils.

## Introduction

### Iron Acquisition by Rice

Iron (Fe) is a transition metal essential for the survival of virtually all living organisms. In plants, Fe participates in several important metabolic processes such as photosynthesis, chloroplast development, chlorophyll biosynthesis, electron transport, and redox reactions (Marschner, 1995). Graminaceous plants, including rice, acquire Fe from soil by chelation (strategy II) method (Römheld and Marschner, 1986). Rice synthesizes the Fe chelator 2′-deoxymugineic acid (DMA), a phytosiderophore of the mugineic acid (MA) family (Takagi, 1976), and secretes it into to the rhizosphere *via* the MA transporter (TOM1) (Nozoye et al., 2011). The secreted DMAs chelate and solubilize insoluble Fe (III) in the rhizosphere to form Fe (III)-DMA complexes. These Fe complexes are taken up into root cells by yellow stripe 1 (YS1) and yellow stripe-like (YSL) transporters, *OsYSL15* (Curie et al., 2001; Inoue et al., 2009). Rice is typically grown in submerged paddy field conditions, where highly soluble ferrous ion (Fe^2+^) is abundant. Under such conditions, unlike other gramineous plants, rice directly takes up ferrous ions *via* iron-regulated transporter 1 and 2 (*OsIRT1* and *OsIRT2*) (Ishimaru et al., 2006) together with phytosiderophores-based Fe uptake.

### Iron Toxicity and its Damage to Rice Plants

The Fe^2+^ ions are abundant in paddy fields and absorption of them by rice roots causes severe Fe toxicity. The rhizosphere of acid sulfate soils with pH less than 5 contains the massive amount of Fe^2+^ about 10–2,000 mg kg^−1^ (da Silveira et al., 2007). The Fe^2+^ ions are highly soluble in water (*Ksp* = [Fe^2+^] [OH^-^]^2^ = 8 × 10^-16^) compared to ferric ions (Fe^3+^) (*Ksp* = [Fe^3+^] [OH^-^]^3^ = 1 × 10^-36^) at 25°C (Stumm and Lee, 1961). Thus, Fe^3+^ ions merely dissolve in water (~1 × 10^-9^ mol L^-1^ at pH 5) while Fe^2+^ ions can dissolve proficiently (~800 mol L^-1^ at pH 5). Therefore, it states that all Fe^2+^ ions which exist in soil can be soluble in low pH (pH 5 or below). Among soil types, mainly acid sulfate soil, acid clay soil, and peat soil cause Fe toxicity (Becker and Asch, 2005). Acid soils occupy approximately 3,950 million hectares or 30% of the total landmass, representing more than 50% of potentially arable land (von Uexküll and Mutert, 1995). Thus, Fe toxicity is one of the major determinants of crop yield and quality, particularly in China, India, and Southeast Asia (NRCS, 2005; Mahender et al., 2019), west and central Africa (Gridley et al., 2006), and Brazil (Crestani et al., 2009).

**Figure 1:**
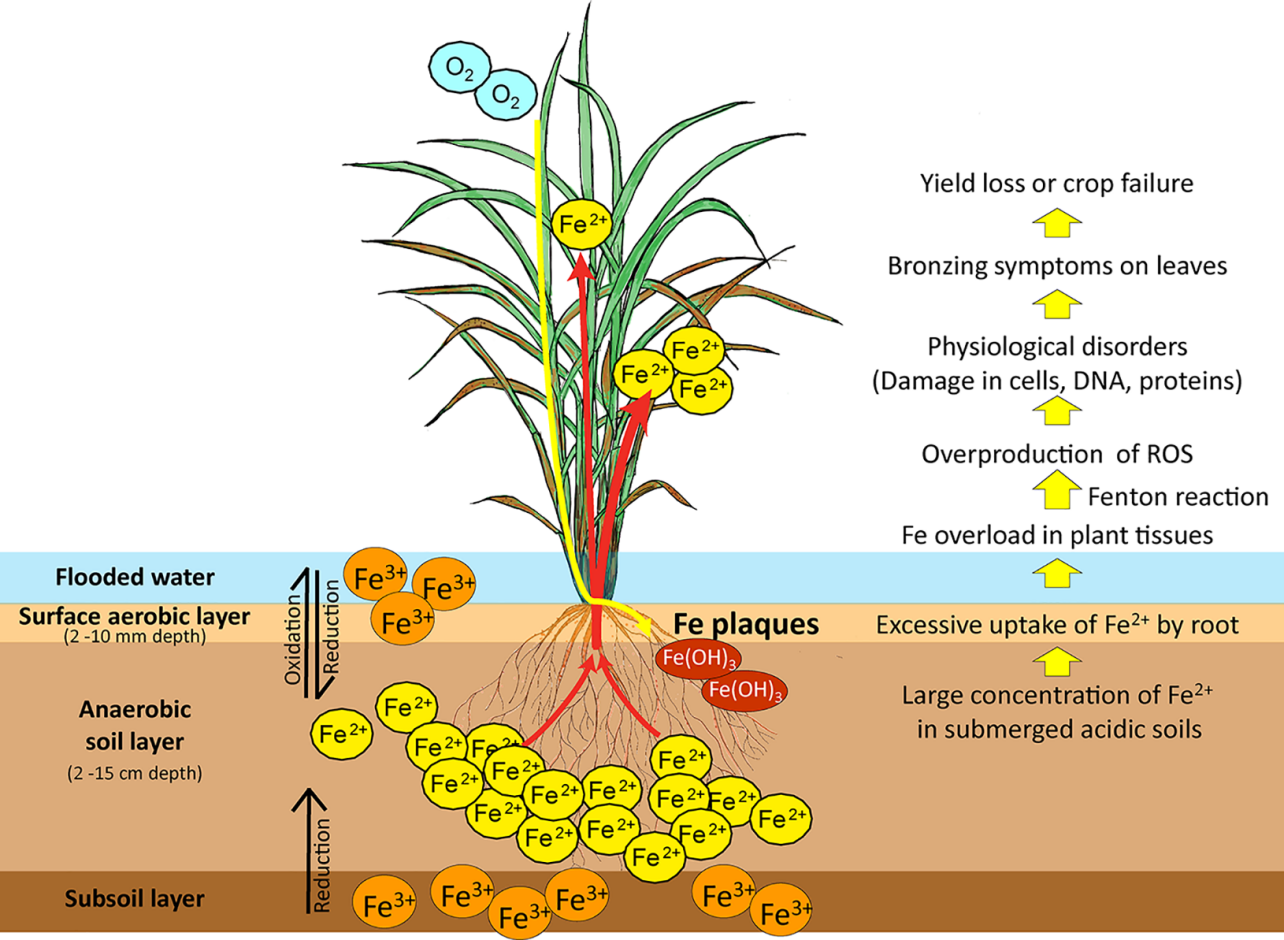
Iron reduction in submerged low-pH soils and the effects of Fe toxicity on rice plants. In submerged soils with anaerobic and low pH conditions, ferric ion (Fe^3+^) is reduced to the more soluble ferrous ion (Fe^2+^). Excess ferrous ion is absorbed by the roots and transported by the xylem to the leaves, causing Fe overload in plant tissues. Excessive Fe accumulation in plant tissues causes overproduction of ROS by the Fenton reaction. These damage cellular structures, membranes, DNA, and proteins and impair physiological processes. Fe toxicity causes bronzing symptoms in leaves, followed by loss of rice yield or complete crop failure. Fe^3+^, sparingly soluble ferric ion; Fe^2+^, soluble ferrous ion; ROS, reactive oxygen species. Red arrows: Fe uptake and transport by rice; Yellow arrow: Rhizospheric oxidation of Fe^2+^ to Fe^3+^ by oxygen transport from shoot to root, leading to formation of Fe plaques on the root surface.

Iron overload in plants causes tissue damage and disrupts cellular homeostasis. The sequential effects of Fe toxicity in rice plants are shown in **Figure 1**. Ferrous toxicity inhibits cell division and elongation of the primary roots and subsequently the growth of lateral roots (Li et al., 2015). High Fe absorption by the roots and its transport into the leaves by xylem *via* the transpiration stream lead to cellular Fe overload in plant tissues (Briat and Lobréaux, 1997).

Free Fe ions caused by Fe overload has a high affinity to bind with oxygen, leading to high oxygen tension and excess Fe accumulation in plant tissues. In plants, the Fe solubility varies in different cellular compartments and their pH is one of the influential factors. For examples, in typical xylem sap at pH 5.5 (Ariga et al., 2014), the solubility of Fe^3+^ is ~3 ×10^-10^ mol L^-1^ and that of Fe^2+^ is ~80 mol L^-1^; in the apoplast at pH 6 (Geilfus, 2017), the solubility of Fe^3+^ is ~1 × 10^-12^ mol L^-1^ and that of Fe^2+^ is ~8 mol L^-1^; in the cytosol at pH 8 (Martinière et al., 2013), the solubility of Fe^3+^ is 1 × 10^-18^ mol L^-1^ and that of Fe^2+^ is ~8 × 10^-4^ mol L^-1^. The Fe^2+^ ions can be oxidized to Fe^3+^ ions, and then those Fe^3+^ ions can readily be precipitated in most of the plant tissues where it becomes immobilized, less reactive, and thus less cellular toxicity. On the other hand, Fe^2+^ ions exist as a highly soluble and reactive form in the plant tissues with Fe overload and even numerous Fe^2+^ ions might exist as a free form in the cytosol if without any chelation or isolation, causing toxic reactions such as Fenton reaction. The Fenton reaction causes overproduction of reactive oxygen species (ROS), particularly the cytotoxic hydroxyl radical (^•^OH) (Becana et al., 1998; Thongbai and Goodman, 2000). Plant Fe toxicity disturbs photosystem II and elevates the cytochrome b6/f content of thylakoids, lowering the photosynthetic rate and accelerating oxygen production (Suh et al., 2002). Fe overload in chloroplasts results in oxidative damage caused by ROS (Balk and Schaedler, 2014). This leads to irreversible damage to cellular structures, membranes, DNA, and proteins (Briat et al., 1995; Stein et al., 2009a). Fe excess in cells impairs biological processes and leads to bronzing symptoms on leaves as a result of cell death. Leaf bronzing induced by Fe toxicity is closely associated with yield loss (Wu et al., 2014).

In this review, we summarize recent progress in the physiological and molecular mechanisms of Fe toxicity in rice, as well as its strategies to maintain Fe homeostasis.

## Physiological and Molecular Defense Mechanisms Against Fe Toxicity

**Figure 2:**
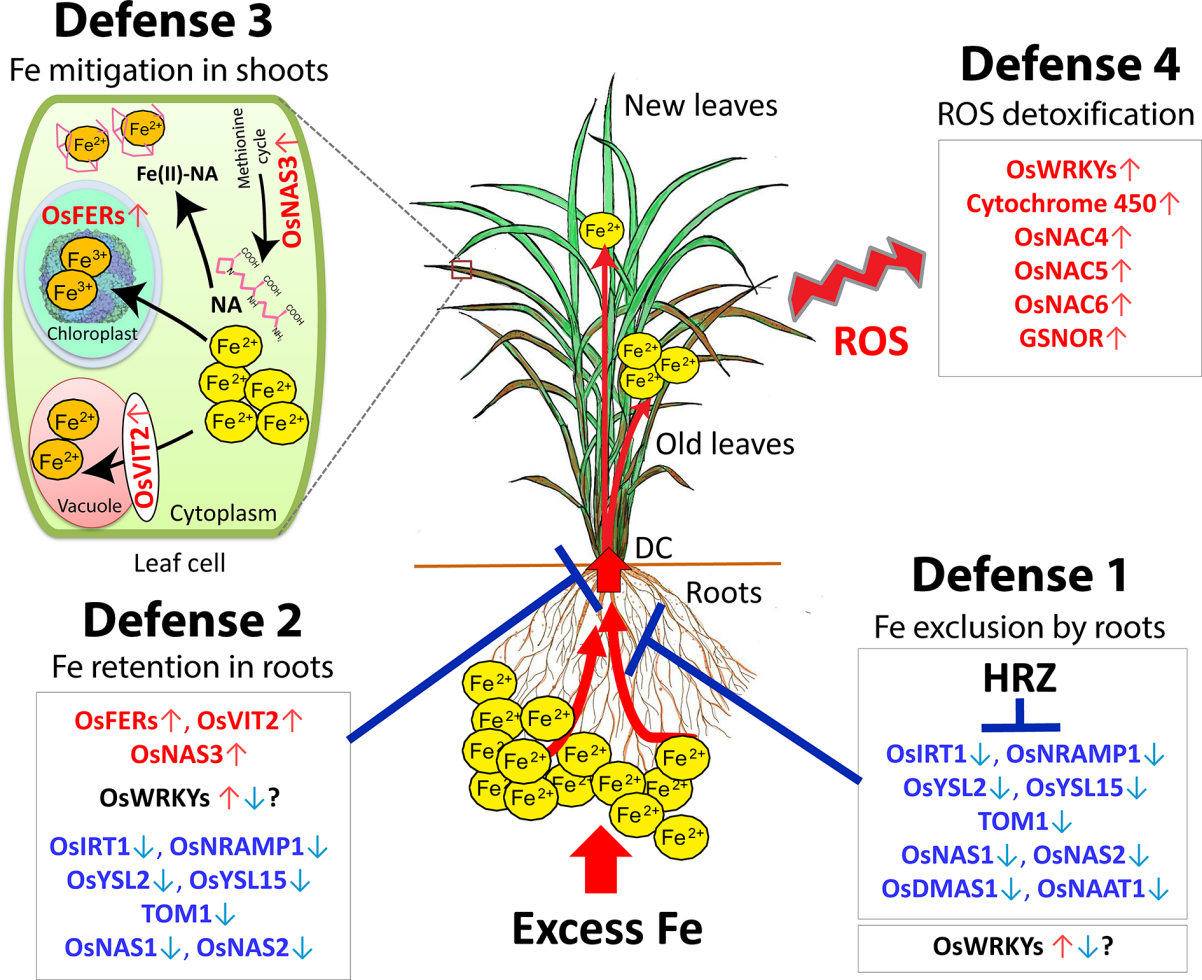
Hypothetical model of the four defense mechanisms of rice against excess Fe. Defense 1: Fe excess tolerance by Fe exclusion in the roots. Defense 2: Fe-excess-tolerance by Fe retention in root and avoidance of Fe translocation to shoot. Defense 3: Fe excess tolerance by Fe compartmentalization in the shoot. Defense 4: Fe excess tolerance by ROS detoxification in the plant. DC, Discrimination center; NA, nicotinamine. Red letters, highly induced genes; Blue letters, highly suppressed genes. This figure is modified from the Supplemental figure of Aung et al., 2018b. The ferritin image was provided by Dr. David S. Goodsell (Scripps Research Institute, La Jolla, CA) and the RCSB PDB.

Rice responds to Fe toxicity by means of four defense mechanisms (Defense 1, 2, 3 and 4). A model of the mechanisms of tolerance to excess Fe is shown in **Figure 2**.

### Defense 1 (Fe Exclusion from Roots)

Under Fe excess condition, rice plants employ a defense mechanism of Fe exclusion, a root-based tolerant mechanism through inhibition of root Fe uptake. Rhizospheric oxidation of Fe^2+^ to Fe^3+^ by oxygen transport from shoots to roots leads to the formation of Fe plaques on the root surface due to Fe^3+^ precipitation, which acts as a barrier to the uptake of excess Fe^2+^ into root tissues (Becker and Asch, 2005). Tolerant genotype with Fe excluder may display the high formation of Fe plaque on the root surface (Mahender et al., 2019). Together with a larger diameter of the pith cavity of the shoots, the genotype with Fe excluder has high root oxidation power by an increase in aerenchyma volume and the number of lateral roots that favors the internal oxygen movement (Wu et al., 2014). Silicon supply mitigates the effect of Fe toxicity on rice by reducing Fe uptake (Ma and Takahashi, 1990). Also, its supply can strengthen the casparian bands in the root epidermis, which serves as a diffusion barrier and inhibits Fe flux into roots under excess Fe (Hinrichs et al., 2017; Becker et al., 2020). Therefore, rice genotypes with enhanced ability to exclude Fe such as high root oxidation power, large aerenchyma, several lateral roots, and strong casparian bands may be superior in Fe excess tolerance.

Microarray analyses have indicated that the expression of Fe uptake- and transport-related genes, such as *OsIRT1*, *OsIRT2*, *OsYSL2*, *OsYSL15*, and *OsNRAMP1*, is highly suppressed in roots in the presence of mild to high Fe excess levels (Aung et al., 2018b). high Fe excess level (Quinet et al., 2012), or very high Fe level (Finatto et al., 2015), suggesting that rice prevents Fe uptake and transport under Fe excess stress. Moreover, the expression of the genes involved in the biosynthesis of MAs, such as *OsNAS1*, *OsNAS2*, *OsNAAT1*, and *OsDMAS1*, is highly suppressed in roots, suggesting that plants restrain DMA release into the rhizosphere under excess Fe (Aung et al., 2018b). Therefore, rice utilizes defense 1 Fe exclusion to prevent Fe uptake by roots under Fe excess condition (**Figure 2**).

### Role of HRZ in Fe Excess Tolerance (in Defense 1)

The HRZ, a Hemerythrin motif-containing Really Interesting New Gene- and Zinc-finger protein, is one of the key Fe homeostasis regulators. Kobayashi et al. (2013) identified two Fe-binding ubiquitin ligases, *OsHRZ1 *and *OsHRZ2*, which negatively regulate Fe deficiency response in rice. *HRZ*-knockdown rice lines are hypersensitive to Fe toxicity, demonstrating severe growth defects and leaf bronzing since even under mild Fe excess conditions (Aung et al., 2018a). In addition, disruption of HRZ causes 5- to 10-fold hyperaccumulation of Fe in leaves, and the expression of Fe uptake- and transport-related genes including *OsIRT1*, *OsYSL2*, *OsYSL15*, *TOM1*, *OsNAS1*, *OsNAS2* and *OsIRO2* is severely elevated in roots of knockdown rice subjected to Fe excess stress (Aung et al., 2018a). Therefore, HRZ is an important protein to protect plant cells from Fe toxicity and maintain Fe homeostasis in plants under Fe excess by repressing the genes involved in Fe uptake and translocation (Aung et al., 2018a, **Figure 2**).

Interestingly, HRZ expression is highly induced in response to Fe deficiency (Kobayashi et al., 2013). However, several Fe-uptake genes are strongly induced in that condition. These results suggest that Fe uptake mechanism by plant should be strictly regulated, and thus *HRZ* is ready to regulate like a break to Fe uptake genes since under Fe deficiency condition. The hemerythrin domains of HRZs, likewise its homolog BRUTUS (BTS) in *Arabidopsis* (Selote et al., 2015; Hindt et al., 2017), are predominantly bound by Fe and zinc (Zn) (Kobayashi et al., 2013), and direct binding of the Fe to the key regulators including HRZ or BTS, may be responsible for intracellular Fe-sensing and signaling events (Kobayashi, 2019). Thus, it is conceivable that HRZ may play a major role as an Fe-binding sensor in the earliest response system to excess Fe.

In addition to defense 1 in roots, HRZs might have additional roles in other tissues or organs under Fe excess condition. Further studies are desirable to clarify HRZ-mediated regulations in other defenses.

### Defense 2 (Fe Retention in Roots and Suppression of Fe Translocation to Shoots)

Rice can retain Fe in the root tissues and decrease Fe translocation from roots to shoots (Tadano, 1975) to avoid excessive Fe accumulation in leaves and maintain homeostasis (Becker and Asch, 2005; da Silveira et al., 2007). In the Fe-toxic field experiment, the tolerant genotype (EPAGRI 108) retains a higher Fe concentration in root symplast (7 mg g^−1^ dry weight) compared to the sensitive genotype (BR IRGA 409 with 1.93 mg g^−1^ dry weight) (Stein et al., 2014). Moreover, the tolerant genotype precipitates approximately 94% of the Fe in its root apoplast during Fe excess. Thus, tolerant genotypes likely to cope with an elevated intracellular and apoplastic Fe concentrations in roots. Hence, protecting aboveground parts from excessive Fe overload is crucial in determining plants’ tolerance level.

In graminaceous plants, various metals absorbed by roots accumulate in the discrimination center (DC) in the basal part of the shoot, and the DC regulates mineral distribution to other plant parts (Mori, 1998). Protecting shoots, particularly the newest leaf, from Fe toxicity is important for the survival of rice plants, and the DC preferentially transports Fe to old leaves rather than new leaves (Aung et al., 2018b). Therefore, the DC may play an important role in Fe partitioning to aerial parts of plants and also act as a barrier to root-to-shoot transport of excessive Fe. Furthermore, Fe transport-related genes such as *OsIRT1*, *OsYSL2*, *OsTOM1*, *OsNRAMP1*, and *OsYSL15* are highly suppressed in the DC, as in roots under Fe excess, suggesting that these genes likely distribute less Fe to shoots, instead retaining Fe in roots and the DC (**Figure 2**).

### Defense 3 (Fe Compartmentalization in Shoots)

When defense 1 and defense 2 systems are insufficient, plants may allow Fe transport from the roots through DC for the subsequent avoidance of Fe toxicity by Fe compartmentalization, disposal, or storage inside shoots. In fact, some tolerant genotypes accumulate more Fe in aerial shoots, thus maintaining healthy shoot growth (Onaga et al., 2013). Indonesian rice genotypes, such as Siam Saba, Mahsuri, Margasari, and Pokkali, reportedly exhibit shoot-based tolerance, as evidenced by a high shoot Fe concentration, reduced leaf bronzing, and a high grain yield (Nugraha et al., 2016).

Notably, the Fe concentration in roots increased nine-fold in the presence of 20-fold Fe excess treatment and then reached to constant at higher Fe treatments (Aung et al., 2018b). By contrast, the concentration in shoots increased in proportion to the level of Fe excess. Therefore, rice has a capacity to retain a limited amount of Fe in root tissues. At Fe levels greater than that can be coped with by defense 2, rice translocates excess Fe from roots to shoots. Interestingly, the higher the Fe excess levels, the more Fe was preferentially translocated to old leaves, which showed bronzing earlier than new leaves. The citrate efflux transporter, *OsFRDL1* is required for root-to-shoot Fe translocation and its expression in nodes is required for Fe distribution to rice grains (Yokosho et al., 2016). The expression of *OsFRDL1* and its homolog *OsFRDL2* is upregulated in roots and the DC at high Fe levels by microarray analyses (Aung et al., 2018b). Thus, *OsFRDL1* and *2* may be important in dividing Fe in DC under Fe excess, presumably for distributing Fe to old leaves than new leaves. To prevent Fe excess-mediated damage to new leaves, rice plants may have a mechanism to partition excess Fe to old leaves to prevent damage to the newest leaves.

### Role of Ferritin and *OsVIT2* in Fe Excess Tolerance (in Defense 2 and 3)

The Fe^2+^ ions, once in the cells, can be associated into proteins or stored in cellular compartments such as ferritins and vacuoles. Ferritin is a ubiquitous Fe storage protein that stores up to 4,000 Fe atoms in a complex (Theil, 2003). Ferritin genes are transcriptionally induced in response to excess Fe in both monocot and dicot plants (Stein et al., 2009b; Briat et al., 2010). By microarray analyses, the rice ferritin genes, *OsFER1 *and *OsFER2*, are strongly upregulated in roots and shoots by excess ferrous treatments (Quinet et al., 2012; Finatto et al., 2015; Aung et al., 2018b). Therefore, excess Fe is isolated into ferritin in a safe and bioavailable form by *OsFER1* and *OsFER2* in response to Fe overload as a part of defense mechanism (Briat and Lobréaux, 1997; Silveira et al., 2009).

Vacuoles are important cellular organelle for Fe isolation to prevent cellular toxicity. The vacuolar Fe transporters, *OsVIT1* and *OsVIT2*, are responsible for the sequestration of Fe into vacuoles *via* the tonoplast (Zhang et al., 2012). *OsVIT1* is less responsive to Fe abundance (Zhang et al., 2012) and its expression is suppressed in all tissues mainly in roots (Aung et al., 2018b). On the other hand, *OsVIT2* is highly responsive to Fe abundance (Zhang et al., 2012) and its expression is strongly upregulated in all tissues subjected to excess Fe (Quinet et al., 2012; Finatto et al., 2015; Aung et al., 2018b). Therefore, *OsVIT2* might be important for the sequestration of excess Fe into vacuoles in a non-toxic form.

In defense 2 and 3, plants allow internal Fe distribution or Fe sequestration in roots and shoots. To detoxify excess Fe in other compartments or tissues, plants transform excess Fe into non-toxic form by accumulation in ferritin and/or disposal of it in a vacuole *via OsVIT2*, or by exclusion from the symplast and efflux into the apoplast (**Figure 2**).

### Role of *OsNAS3* in Fe Excess Tolerance (in Defense 2 and 3)

Nicotianamine (NA) is a major chelator of metal cations in higher plants. It is a chelate compound involved in Fe transport within the plant body. Among the three NA synthase genes in rice, *OsNAS1* and *OsNAS2* are involved in Fe deficiency response (Inoue et al., 2003). On the other hand, the expression of *OsNAS3* is strongly induced in response to Fe excess in various tissues including root, DC, stem, and old and newest leaves (Aung et al., 2018b). Also, the expression of the methionine cycle-related genes is not completely suppressed in Fe excess roots and other tissues. Thus, it is thought that methionine cycle works under excess Fe as well in producing NA along with *OsNAS3* **(** **Figure 2** **)**.

Promoter-*GUS* (β-glucuronidase) analyses have revealed high-level *OsNAS3* expression in the vascular bundle, epidermis, and exodermis of the root, stem, and old leaf under Fe excess (Aung et al., 2019). Moreover, disruption of *OsNAS3* by T-DNA knockout increases sensitivity to excess Fe, reduces growth, causes severe leaf bronzing, and promote Fe accumulation in leaves. These findings provide the evidence that *OsNAS3* is crucial to mitigate excess Fe. NA synthesized by *OsNAS3* contributes to chelate excess Fe in roots and shoots, then it can be stored as a safe form since Fe chelated with NA does not cause the Fenton reaction. It may also protect the photosynthesis and water loss in leaves during Fe excess stress. NA involves in Fe-buffering to maintain Fe homeostasis under Fe excess (Pich et al., 2001) and plays a role in scavenging Fe and protecting the cells from oxidative damage (von Wirén et al., 1999). Therefore, it suggests NA synthesized by *OsNAS3* chelates excess Fe and contributes efficient Fe translocation and sequestration, exhibiting its important role in mitigation of excess Fe in defense mechanisms 2 and 3, and in Fe excess tolerance in rice.

### Defense 4 (ROS Detoxification)

The fourth defense mechanism is Fe inclusion with tolerance to cellular Fe overload and damage in leaves, which is mediated by enzymatic detoxification in the symplast (Becker and Asch, 2005; Stein et al., 2009a), scavenging of ROS by antioxidants (e.g., ascorbic acid and reduced glutathione) (Fang et al., 2001; Gallie, 2013), and protection from ROS damage by the action of antioxidant enzymes (e.g., superoxide dismutase, peroxidase, and catalase) (Bode et al., 1995; Fang and Kao, 2000). Cultivation with various Fe excess intensities and microarray analyses revealed that the molecular defense mechanism of the defenses 1, 2, and 3 works since mild to moderate Fe excess conditions that do not seriously affect rice growth yet. However, the genes contribute to defense 4 work in molecular level in much severe Fe excess condition, which causes severe leaf bronzing and remarkably reduces the plant growth (Aung et al., 2018b).

Under severe Fe excess, plants induce the expression of genes associated with oxidative stress, oxygen and electron transfer, and those encoding cytochrome P450 family proteins (Quinet et al., 2012; Aung et al., 2018b), or transcription factors related to cell death and stress responses (*OsNAC4*, *OsNAC5*, and *OsNAC6*) (Finatto et al., 2015; Aung et al., 2018b). to alleviate the damage caused by excess Fe-induced ROS and other abiotic stresses caused by Fe excess.

Li et al. (2019) reported that *S-nitrosoglutathione-reductase* (GSNOR) promotes root tolerance to Fe toxicity by inhibiting Fe-dependent nitrosative and oxidative cytotoxicity in plants *via* the nitric oxide pathway. In *Arabidopsis*, knockout mutants display a root growth defect in the presence of high Fe (250 µM), demonstrating that GSNOR is important for root tolerance to Fe toxicity. Then, WRKY transcription factors are important superfamilies and key regulators of several processes associated with numerous abiotic and biotic stress tolerance in plants. Most WRKYs genes are critical in regulation of various contrasting stresses simultaneously, and also involved in ROS defense mechanism to various stresses including Fe excess stress (**Figure 2**).

### Possible Working Model of Four Defenses

Given the roles of each Fe excess defense mechanism, the hypothesis is outlined in the model shown in **Figure 2**. Whenever the plant receives the Fe excess stress, defense 1 (Fe exclusion) works to inhibit root Fe absorption by the suppression of Fe uptake genes. This defense might protect well the plant in conditions of weak Fe toxicity. When Fe excess is more sever, defense 1 may no longer prevent the roots from excessive Fe uptake because some metal transporters also absorb Fe at the same time in the process of absorbing other essential metals such as Zn, copper (Cu) or manganese (Mn). In such a condition, defense 2 (Fe retention in roots and suppression of Fe translocation to shoots) works by accumulating Fe in a non-toxic form in safety compartments of roots that leads an increase in root Fe concentration, and also prevents the root-to-shoot Fe translocation by the suppression of the related genes. It is likely that defense 1 and 2 mechanisms constitutively drive since prior to the onset of visual damage in leaves and throughout the stress from low to severe Fe excess levels.

When defense 2 is insufficient to sequester excess Fe in root tissues, the excess Fe is translocated from root to shoot. In this case, defense 3 (Fe compartmentalization in shoots) begins to employ the functions of chelation, isolation and/or sequestration of excess Fe in a safe form in the shoot that leads an increase in shoot Fe concentration. Lastly, when free divalent Fe cannot be completely sequestered in safety form under severe Fe conditions, it becomes largely accumulated in the cytosol and causes Fenton reaction that generates ROS stress. In this condition, the corresponding genes in defense 4 (ROS detoxification) are induced to mitigate ROS stress in plants. Overall, these four defense mechanisms might work cooperatively and consecutively for Fe detoxification in rice depending on the degree of Fe excess progression.

## Future Prospects For Crops Tolerant To Excess Fe

### Gene Response and Regulatory Mechanism to the Fe Toxicity in Plants

There has been progress in our understanding of Fe excess defense and tolerance mechanisms in plants. Fe deficiency response and its regulation network in plants is well characterized and widely understood (Kobayashi et al., 2019). However, the Fe excess-signaling factor, transcription factors and regulatory genes conserved in the molecular mechanism of the Fe toxicity response are not well identified yet. Some genes involved in defense system may start to work after receiving a direct Fe excess signal mediated by ROS. Also, HRZ-mediated regulation to Fe-homeostasis-related genes is crucial in Fe excess as described above. But the other potent regulators and the genes participated in the Fe-excess responsive pathway are still largely unknown.

Viana et al. (2017) reported that the *cis*-regulatory elements involved in responses to abiotic stresses, such as light and salicylic acid pathway, participate in the molecular signaling involved in the response to Fe excess. However, promoter analyses of the functional *cis*-acting elements for the core Fe excess-responsive in rice still needs to be identified. Some bHLH transcription factors have important roles for Fe homeostasis in plants. OsIRO3 (OsbHLH063) acts as a negative regulator of the Fe deficiency response in rice (Zheng et al., 2010) and also involves in shoots-to-roots signal transmission which is important to prevent Fe toxicity in plants (Wang et al., 2020). Moreover, some WRKY transcription factors may also involve in Fe homeostasis in plants. Ricachenevsky et al. (2010) firstly reported the Fe-excess induced transcription factor, *OsWRKY80* in rice, which is also regulated by the dark-induced senescence- and drought- stress. In that study, *OsWRKY80* transcript level was increased up to threefold in roots and shoots after 9 days of Fe excess. Furthermore, microarray analyses have yielded 19 upregulated WRKYs in Fe-excess rice (Finatto et al., 2015). Then, *OsWRKY55-like*, *OsWRKY46*, *OsWRKY64*, and *OsWRKY113* are upregulated in an Fe-sensitive genotype, and *OsWRKY55-like* may be involved in the early stress response (Viana et al., 2017). Considering these results, some WRKY genes may also involve in defense mechanisms 1, 2 and 3 in addition to defense 4 (**Figure 2**). Therefore, transcription factors, such as some bHLHs and WRKYs, may involve in Fe excess response mechanism of the plants. Further comprehensive studies are required for the discovery of their specific roles in Fe excess. Therefore, exploration of Fe-excess responsive *cis*-acting elements, transcription factors, other potent regulators and candidate genes becomes a prerequisite for the deeper understanding of the mechanism of the excess Fe response and the development of rice varieties tolerant to Fe toxicity.

### Developing Fe Excess Tolerant Rice *via* Molecular Breeding

Based on the tolerant rice lines selected by screening, the introduction of target genes into tolerant genotypes will increase further tolerance to Fe toxicity stress. As described above, the previous- and new-finding candidate genes encoding ferritin, VIT, NAS3, HRZ, GSNOR, and WRKYs may contribute to Fe excess tolerance in plants. In addition to these genes, there is a number of genes associated to the response to excess Fe; for example, overexpression of *Arabidopsis* Fe transporter, *AtNramp1*, leads to resistance to Fe toxicity (Curie et al., 2000). Use of these promising target genes and/or other candidate Fe homeostasis-related genes in molecular-breeding approaches, such as marker-assisted breeding, transformation, or genome editing methods, will facilitate the development of genotypes that are well adapted to Fe toxicity and contribute to increasing rice productivity in hotspot sites of soils with excess Fe. In addition, Fe excess-responsive genes have potential for addressing the global issue of Fe biofortification in grains (Masuda et al., 2020).

## Conclusion

Understanding of the molecular mechanisms of Fe excess tolerance in rice is essential for varietal development. There is a progress in discovery of candidate genes involved in the physiological and molecular response of rice to Fe overload, and their roles in Fe homeostasis, Fe detoxification, and Fe excess tolerance are becoming clear. These genes could be promising targets of genetic resources for the development of rice genotypes with increased Fe toxicity tolerance by breeding and to increase rice productivity in regions prone to Fe toxicity to meet the food demand for increasing population.

## Author Contributions

MSA and HM designed and wrote the manuscript. MSA led the writing of the manuscript. HM edited and improved the manuscript.

## Funding

This publication was supported by a Grant-in-Aid for Young Scientists (B) (Grant No. 18K14367) from Japan Society for the Promotion of Sciences, JSPS KAKENHI (to MSA).

## Conflict of Interest

The authors declare that the research was conducted in the absence of any commercial or financial relationships that could be construed as a potential conflict of interest.
