# Corrigendum: How Does Rice Defend Against Excess Iron?: Physiological and Molecular Mechanisms

**DOI:** 10.3389/fpls.2020.601527

**Published:** 2020-11-20

**Authors:** May Sann Aung, Hiroshi Masuda

**Affiliations:** Department of Biological Production, Faculty of Bioresource Sciences, Akita Prefectural University, Akita, Japan

**Keywords:** iron excess, rice, OsNAS3, HRZ, OsVIT2, ROS, iron homeostasis, tolerant mechanism

In the original article, there was a mistake in Figure 2 as published. **OsNAS↑** **instead of OsNAS3↑** **in Defense 3**. The corrected **Figure 2** appears below.

**Figure 2:**
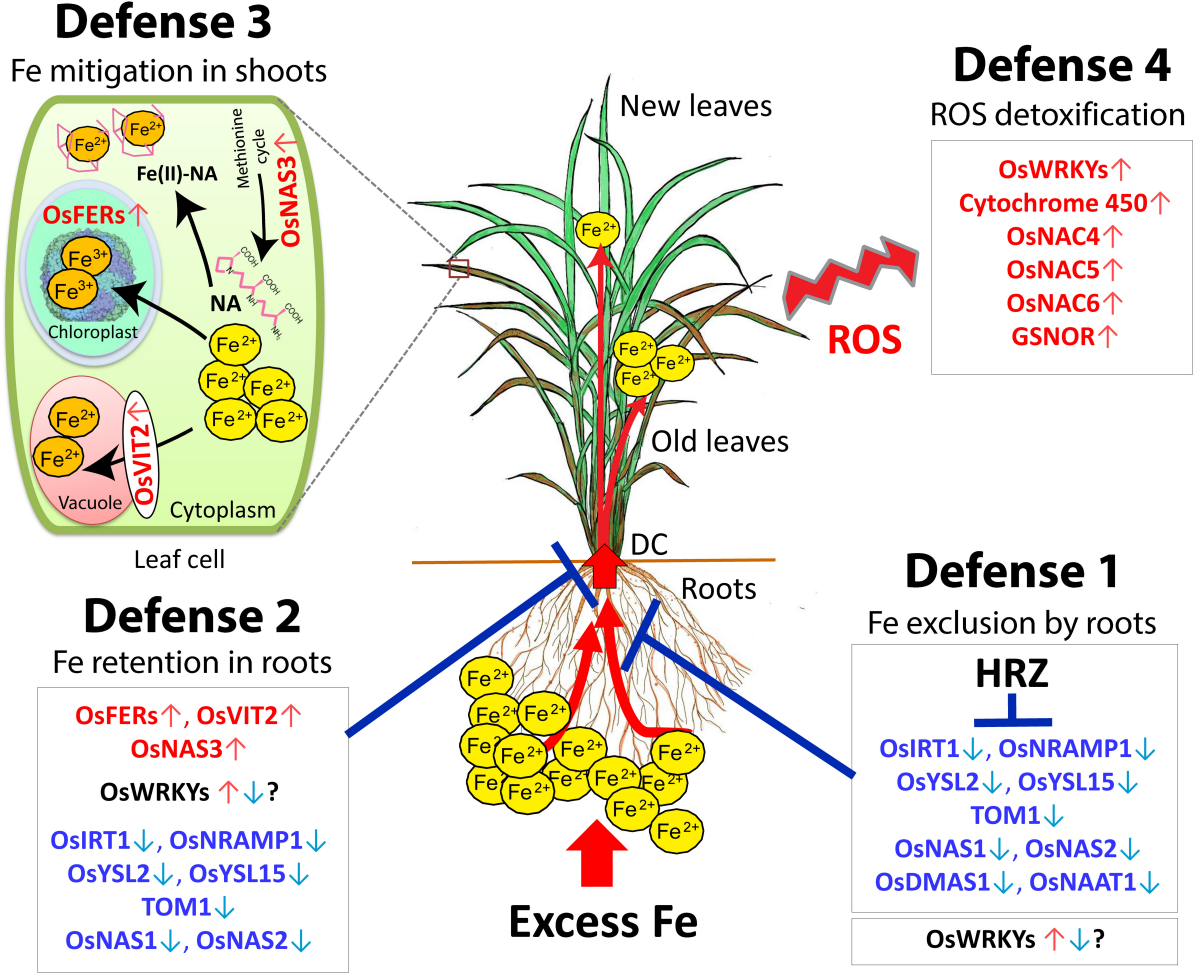
Hypothetical model of the four defense mechanisms of rice against excess Fe. Defense 1: Fe excess tolerance by Fe exclusion in the roots. Defense 2: Fe-excess-tolerance by Fe retention in root and avoidance of Fe translocation to shoot. Defense 3: Fe excess tolerance by Fe compartmentalization in the shoot. Defense 4: Fe excess tolerance by ROS detoxification in the plant. DC, Discrimination center; NA, nicotinamine. Red letters, highly induced genes; Blue letters, highly suppressed genes. This figure is modified from the Supplemental figure of (Aung et al., 2018b). The ferritin image was provided by Dr. David S. Goodsell (Scripps Research Institute, La Jolla, CA) and the RCSB PDB.

The authors apologize for this error and state that this does not change the scientific conclusions of the article in any way. The original article has been updated.
